# Clinical review of stroke care at National District Hospital, Bloemfontein

**DOI:** 10.4102/safp.v65i1.5608

**Published:** 2023-01-05

**Authors:** Selma Smit, Dirk T. Hagemeister, Cornel van Rooyen

**Affiliations:** 1 Department of Family Medicine, Faculty of Health Science, University of the Free State, Bloemfontein, South Africa

**Keywords:** stroke, stroke unit, emergency stroke care, neurological outcome, thrombolysis, rehabilitation

## Abstract

**Background:**

Stroke is a leading cause of morbidity and mortality affecting sub-Saharan Africa. Studies show that dedicated stroke units improve patient outcomes. National District Hospital (NDH) manages strokes, with the potential of becoming a dedicated stroke unit in Bloemfontein, South Africa. The study aimed to describe the clinical characteristics, management and outcomes of patients presenting with stroke at NDH.

**Methods:**

In this retrospective descriptive study, emergency department registers were used to identify patients presenting with symptoms of a stroke between 01 January 2019 and 31 March 2019. Relevant data were extracted from hospital files.

**Results:**

Of the 106 identified patients, 53 were included in the study. The median age was 61 years (range 28–89 years), with an almost equal split between genders. The most common risk factor was hypertension (81.3%). The median time from symptom onset to presentation at NDH was 9 h. No patient received thrombolysis. One patient received neurosurgical intervention. The most prescribed secondary preventative drugs were antihypertensive medication, statins, anticoagulation and antiretroviral therapy. Half (52.8%) of the patients received rehabilitation as in-patients. Final diagnoses were ischaemic strokes (26/53, 49.0%), transient ischaemic attacks (10/56, 22.7%) and haemorrhagic strokes (6/56, 13.6%). The 6-month post-infarct mortality rate was 37.5%.

**Conclusion:**

Patient outcomes were comparable to similar South African studies. Time delays in stroke management remain a major obstacle. Identified action points include community education, improving emergency medical services and establishing a dedicated stroke unit.

**Contribution:**

This study underlines the importance of stroke and cardiovascular disease prevention and stresses the value of establishing dedicated stroke units.

## Introduction

Stroke, or cerebrovascular accident, is a common and often devastating disease with a high societal cost. The high burden of cerebrovascular disease in South Africa makes it an important topic for research. A thorough assessment of the current situation can help guide clinical governance decisions to advance healthcare services in an effective, equitable and financially prudent manner.

The American Heart Association and the American Stroke Association released a consensus document^1^ in 2019 on the updated definition of stroke, intending to include both clinical and tissue criteria into the definition. The term ‘stroke’ or ‘central nervous system (CNS) infarction’ can be understood as follows:

Central nervous system infarction is defined as brain, spinal cord, or retinal cell death attributable to ischemia, based on neuropathological, neuroimaging, and/or clinical evidence of permanent injury. Central nervous system infarction occurs over a clinical spectrum: Ischemic stroke specifically refers to central nervous system infarction accompanied by overt symptoms, while silent infarction causes no known symptoms. Stroke also broadly includes intracerebral hemorrhage and subarachnoid hemorrhage.^1^

Stroke is one of the most common causes of death globally, but even more so in developing countries.^2^ Approximately, 6.55 million lives were lost worldwide in 2019 because of strokes (both ischaemic and non-ischaemic), with a disproportionate number of those deaths occurring in sub-Saharan Africa and Asia.^2^ Stroke-related morbidity and mortality will likely increase further in the future because of the effects of population growth and ageing.^2,3^ Death, however, is not the only poor outcome to be considered when discussing strokes: disability is a significant permanent consequence affecting some patients (and their families) who survive the neurological insult.

The timing of medical intervention in the management of stroke is an important factor in improving neurological outcomes and decreasing mortality, particularly the timing of intravenous thrombolytic therapy.^4,5^ Recent guidelines have set out to extend thrombolytic windows in ischaemic strokes, although the outcomes when giving the thrombolytics within the first 4.5 h remain better than a longer time interval.^4^ Ischaemic strokes account for most (> 80%) of the strokes seen, while haemorrhagic strokes account for most non-ischaemic strokes.

The Framingham Heart Study^6^ has greatly contributed to what is known today as risk factors for a stroke. The two most significant contributors are hypertension (with mean systolic hypertension playing a more prominent role in strokes than diastolic hypertension) and atrial fibrillation. These two factors greatly increase a patient’s risk of stroke, even when correcting for non-modifiable risk factors, such as age and gender. Other risk factors that lead to atherosclerosis also increase the risk for a stroke, such as diabetes mellitus, dyslipidaemia and tobacco smoking.^3,6,7^ Human immunodeficiency virus (HIV) is also an important risk factor for developing a stroke, especially haemorrhagic strokes and often affects younger HIV-positive patients.^8,9^

The South African population has a high prevalence of several risk factors predisposing to strokes – including hypertension, diabetes, HIV/AIDS, tobacco use and obesity. About a third of stroke patients die within 28 days with recent estimates putting the death toll from stroke at about 25 000 deaths per year.^10,11,12^ The incidence of stroke appears to be increasing because of an uptick in non-communicable diseases.^10,11,12^ The impact of stroke on a population cannot be measured by the mortality rate alone, as many stroke survivors continue to live with moderate to severe disability. This has far-reaching effects, not only on the patient’s quality of life but also on the national economy. Bertram et al.^10^ estimated the annual cost of cardiovascular disease and its complications in 2011 to be approximately 13–16 billion Rand – excluding the cost of rehabilitation.

A review of stroke outcomes was carried out in 2015 by Maredza et al.^11^ on a national scale. The researchers used the Modified Rankin Scale (MRS)^13^ to determine the severity of disability after the stroke and found that 58% of stroke victims suffered from some form of disability.

Stroke management should ideally take place in a dedicated stroke unit and be protocol-driven.^14,15,16^ Dedicated stroke units have been found to be cost-effective^17,18^ and could improve patient outcomes.^19^

### Aim

The aim of this study was to describe the clinical characteristics, management and outcomes of patients presenting with stroke at National District Hospital (NDH) in Bloemfontein, from 01 January 2019 to 31 March 2019.

The objective of this study was to describe:

- the presentation at the emergency department, including time since the onset of symptoms, as well as presenting signs and symptoms
- initial management of strokes in the emergency department and in-patient setting at NDH
- patients’ demographic profile
- patient risk factors that may contribute to stroke
- patient outcomes six months after the incident and final diagnosis.

## Methods

### Study design

A retrospective descriptive study was conducted at NDH in Bloemfontein, South Africa. National District Hospital is a level 1 public hospital based in an urban area, servicing patients from the Mangaung Metropolitan area and surrounding small towns. At present, NDH does not have a dedicated stroke unit and therefore there were no clear treatment guidelines, but provides 24-h emergency services.

### Study population and sampling

All patients presenting to NDH emergency department with signs and symptoms suggestive of stroke between 01 January 2019 and 31 March 2019 were included in the sample, retrospectively reviewing their clinical notes in their hospital files. Signs and symptoms used to screen patient files for possible stroke were focal neurological fall-out, first onset seizures and a decreased level of consciousness.

### Measurement

Casualty registers were used to identify patients whose presenting complaints could indicate a stroke. Patient and clinical data were extracted from the hospital files using a datasheet designed by the researcher.

### Pilot study

A pilot study was performed on the first three patients on the casualty register who presented with signs and symptoms of a stroke to the NDH emergency department in December 2018. After the pilot study, the datasheet was adjusted and resubmitted to the ethics committee for approval. The patients in the pilot study were not included in the final sample.

### Data analysis

Data were analysed by the Department of Biostatistics, Faculty of Health Sciences of the University of the Free State using statistical analytics software. Descriptive statistics, namely medians and percentiles, were calculated for continuous data. Frequencies and percentages were calculated for categorical data.

All patient-related information was kept confidential, and no identifiable patient details were used in any form of publication. No informed consent from the patients was required.

### Ethical considerations

Ethical approval was obtained from the Health Sciences Research Ethics Committee at the University of the Free State [UFS-HSD2020/0164/2710-0001]. The Free State Department of Health gave permission to conduct the study.

## Results

### Patient demographics

For the study period, 106 cases were identified for inclusion. Only 66 of the 106 files could be retrieved (because of poor record-keeping practices), and eventually, only 53 patients qualified for inclusion in the study. Reasons for exclusion were diagnoses other than stroke. The median age of patients presenting with a stroke was 61 years (range 29–89 years). Just over half (50.9%) of the patients were females while 49.1% were males. Mangaung Metro residents comprised 84.9% of the patients, while 15.1% were from the surrounding smaller towns. Of the 53 patients, 32 (60.4%) used public ambulance emergency medical services (EMS), 15 (28.3%) used transport with a private car or taxi, while only three (5.6%) used a private ambulance service. Three patients had no data recorded on the method of transportation used.

Patients presented to the emergency department on average 9 h after the initial onset of symptoms. Sixteen (37.2%) patients presented within 4.5 h (the thrombolytic window).^4,5,20^ Seventeen (39.5%) patients presented 24 h or more after the initial onset of symptoms. In this emergency department, about 25–30 strokes are seen per month.

### Clinical characteristics

Pre-existent risk factors for stroke and their frequency are shown in Table 1.

**TABLE 1:** Frequency of pre-existing risk factors for stroke (*N* = 53).

Risk factor	*n*	%
Hypertension	43	81.3
Known vascular disease	12	22.6
Diabetes	9	17.0
Smoking	9	17.0
HIV	8	15.1
Dyslipidaemia	4	7.6
Atrial fibrillation	1	1.9

HIV, human immunodeficiency virus.

Twenty-four (45.3%) patients presented with left-sided hemiplegia, and 23 (43.4%) patients with right-sided hemiplegia. Four (7.6%) patients did not have hemiparesis at the time of presentation. More than half (*n* = 30, 56.6%) of the patients suffered from dysarthria, and 37 (69.8%) patients presented with upper motor neuron facial nerve palsy. The Glasgow Coma Scale (GCS) was recorded in 52 patients, with 26 (50.0%) patients scoring 15/15. Only two (3.9%) patients scored below 8/15 on the GCS, and three (5.7%) patients presented with cerebellar signs. Pupil reaction was observed as equal in 49 (92.5%) patients.

### Management

More than half (*n* = 30, 56.6%), of the patients presenting with signs and symptoms of stroke underwent an elective computerised tomography (CT) scan, 17 (32.1%) an emergency CT scan and six (11.3%) patients received no CT scan. No data were available on why some patients did not receive a CT scan at all.

Interventions included in-patient interventions at NDH (*n* = 28, 52.8%) and transfer to neurosurgery (*n* = 1, 1.9%). None of the patients received thrombolysis. Data on further management were unavailable for 25 (47.2%) patients. Special investigations included an electrocardiogram (ECG) (*n* = 47, 89.7%), echocardiogram (*n* = 10, 18.9%), carotid doppler (*n* = 10, 18.9%) and blood tests (*n* = 17, 32.1%). Secondary preventative measures included statins (*n* = 44, 83.0%), anticoagulants (*n* = 42, 79.3%), antihypertensives (*n* = 40, 75.5%) and antiretrovirals (*n* = 12, 22.6%).

Patients spent a mean time of 11 h and 49 min in the emergency department from the time of presentation until admission (*n* = 29). Time from presentation to ward admission ranged from 45 min to 45 h. The average in-patient length of stay was calculated at 3.94 days (*n* = 26). Patients awaited rehabilitation services on average 40 h and 42 min in the ward; this was calculated from the time of admission until the time recorded by the rehabilitation team in the files. Forty-one (77.4%) patients were admitted to NDH, while 11 (20.8%) were transferred to Pelonomi Tertiary Hospital and one patient was transferred to Universitas Academic Hospital.

### Patient outcomes 6 months after the incident

**FIGURE 1:**
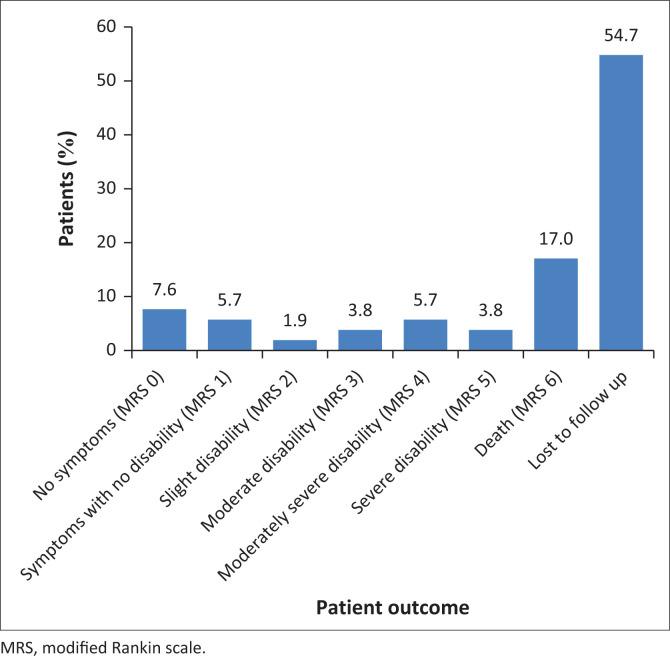
Patient outcomes six months after the initial event (*n* = 53).

Patient outcomes 6 months after the initial event, as recorded in the standard follow-up notes in the patient files, were also assessed using the MRS.^13^ As shown in Figure 1, 29 (54.7%) patients were lost to follow up. Of the remaining 24 patients who had available follow-up notes, nine (37.5%) patients died.

### Final diagnosis

The final diagnosis of 44 patients was recorded. Twenty-six (59.9%) patients suffered an ischaemic stroke, while six (13.6%) patients suffered a haemorrhagic stroke, which did not require thrombolysis. Transient ischaemic attacks made up 10 (22.7%) cases, while one (2.3%) patient had Todd’s paresis, and one (2.3%) patient had an infective intracranial mass (tuberculoma). None of the patients had conversion syndrome or a malignant intracranial mass.

## Discussion

### Demographics

The demographic profile of patients presenting with stroke indicates that most of the patients are middle-aged or elderly, but 11 (20.8%) of the strokes occurred in patients younger than 49 years.^21^ A high percentage (88.9%) of the so-called ‘young strokes’ had either or both hypertension and HIV as a comorbidity. This number of young strokes is significantly higher than the global incidence of strokes in young adults, estimated to be around 10% – 15%.^21^

Males and females were roughly equally affected in this study sample, which is not in keeping with global statistics, which tend to show that men are about 1.5 times more likely to be affected than women.^22^ The reason for this is unclear and likely multifactorial. One of the postulated reasons is a higher prevalence of obesity among South African women versus men.^23^ Men in this study were more likely to be affected at a younger age than women. The median age of stroke was 55 years for men versus 63 years for women. This trend is supported by global data.^22^

The majority (84.9%) of patients resided within the Bloemfontein sub-district, in a roughly 15 km radius from the hospital. The median time from the onset of symptoms to presenting at the hospital was 9 h, with a wide range from less than an hour to 168 h. This delay is worrying, as it reduces the number of patients who potentially qualify for thrombolytic therapy. Sixteen patients did present in time for possible thrombolytics. Still, because of the nature of the referral system to obtain a CT scan at another hospital, not a single patient could be thrombolysed.

Thaddeus and Maine describe the ‘Three Delays Model’,^24^ which would also apply to this situation. The delays are: (1) delay in the decision to seek care, (2) delay in arrival at a health facility and (3) delay in the provision of adequate care.

Patients (and their families) may not realise the seriousness of the symptoms they are experiencing and because most strokes are painless, they might delay seeking help. The South African Guideline for the management of ischaemic stroke and transient ischaemic attack^25^ puts education (of the patients and community) as one of the top priorities to improve knowledge to recognise the signs and symptoms of a stroke. Community education programmes have shown to be successful in decreasing the pre-hospital delay and ultimately improving the prognosis of patients.^26,27,28^

The second delay described is the delay to arrive at the facility. Most patients (60.4%) used public EMS to reach the hospital. The specific time delays involved with the EMS services were not explored as part of this study but could be explored in future studies of this nature. Notably, the three patients who used a private ambulance service arrived within 4 h after the onset of symptoms. The third delay described is the delay in receiving appropriate care at the hospital. Unfortunately, no patients received thrombolytic therapy during the 3-month study period because of the delays discussed here, as well as further delays at the hospital itself.

The referral system is wrought with inefficiencies, such as delays in getting hold of the registrar on call for internal medicine, who needs to discuss the scan with the registrar on call for radiology. In addition, some patients must be transported from one hospital to the other using an overburdened and inefficient EMS system. It is logistically impossible to get a CT scan within 20 min of the patient arriving at the facility because of these problems. For those who received rehabilitation as in-patients, the median time from admission to the initiation of rehabilitation was 40 h and 42 min. This delay could be attributable to patients awaiting a bed in the emergency department and rehabilitation services not being available after-hours or over weekends.

### Management

Patients were triaged according to the South African Triage Scale (SATS) and received priority in the emergency room to be stabilised. All patients had basic vital signs performed (blood pressure, pulse, temperature, respiratory rate, blood glucose and oxygen saturation), and two patients required emergency intubation to secure an airway. Blood tests and ECG were the most common tests performed. Still, not enough patients received ECGs – especially considering that many strokes can be attributed to dysrhythmias, such as atrial fibrillation. Blood pressure and blood glucose management were carried out in patients who required pharmacological support.

Thrombolysis is the backbone of ischaemic stroke management, but as none of the patients in this group received it, this study could not evaluate its administration. One out of the six patients who presented with a haemorrhagic stroke received neurosurgical intervention; the rest were managed conservatively or palliated depending on the severity of the stroke.

All patients admitted to NDH received rehabilitation in the form of physiotherapy and occupational therapy, which is available on site. There was a significant time delay in receiving rehabilitation because of such services being unavailable after hours and over weekends. No data are available for this group of patients on speech therapy, as it is not available on site and only available at Pelonomi Tertiary Hospital.

### Risk factors

According to the World Health Organization, roughly 27.4% of men and 26.1% of women in South Africa have hypertension.^29^ The risk factor profile of the patients presenting with stroke is also in keeping with prior studies on the subject, showing that hypertension is by far the most critical risk factor in the development of strokes.^6,8,9,11,21,25^ Hypertension as the only risk factor was present in 37.7% of cases, while 35.9% of patients had hypertension combined with other risk factors. Only 18.7% of patients who presented with stroke did not have hypertension as a risk factor. Systolic hypertension is of greater concern than diastolic hypertension,^21^ but this study did not differentiate between the two entities. Blood pressure control should be of great priority in primary healthcare, as risk reduction could dramatically decrease the burden of disease.^21,25^

Another major risk factor for the development of stroke (especially thromboembolic strokes) is the presence of atrial fibrillation. Global stroke statistics estimate that 9.3% – 19.0% of ischaemic strokes are because of atrial fibrillation,^6,30^ yet only 1.9% of patients presenting with stroke at our facility had documented atrial fibrillation. This could be because of the lower mean age of the first stroke (as the prevalence of atrial fibrillation increases with increasing age) that the attending clinician missed the diagnosis, or that the episode of atrial fibrillation was intermittent and not present at the time the patient was seen in the emergency department.

Twelve (22.6%) patients presenting with stroke had other manifestations of major target organ damage, such as ischaemic heart disease, peripheral vascular disease or previous strokes. Manifestations of cardiovascular disease often point to widespread atherosclerotic changes in the entire cardiovascular system, not just in a confined area. Patients with one manifestation of atherosclerosis should be initiated on the best medical therapy to decrease their risk of suffering a stroke.^12,18,20^

Human immunodeficiency virus is considered an important risk factor for the development of stroke because of widespread vasculitis and systemic inflammation.^8,9^ In this study population, 15.1% of patients had HIV as comorbidity. This is marginally higher than the overall HIV prevalence for South Africa, which is estimated at 13%.^31^ However, the HIV percentage in our study population was much lower than the 25.5% reported in 2018 after the 2017 survey.^32^

Diabetes, disorders of lipid metabolism and tobacco smoking were also recorded and are known risk factors for the development of cardiovascular disease.^3,6,7^ In this study population, 17.0% of the patients were diabetic, 17.0% admitted to tobacco smoking and 7.6% had known dyslipidaemia. Metabolic risk factors play a major role in the development of atherosclerotic disease, which contribute to developing both ischaemic and haemorrhagic strokes.^3,6,7^

Stroke prevention should be a key priority in the district health plan and strengthening the primary healthcare system is essential to improve outcomes for stroke patients. Stroke prevention can be divided into five stages: primordial prevention, primary prevention, secondary prevention, tertiary prevention and quaternary prevention.^33^

Primordial prevention would include laws and policies to improve the social and environmental conditions that contribute to the disease. Sugar tax has already been implemented to try and address the obesity epidemic and decrease cardiovascular risk factors.^34^ Increased taxation on tobacco products also falls into this category. Primary preventative measures include preventing hypertension, dyslipidaemia and diabetes and non-pharmacologic strategies and lifestyle changes, such as stopping smoking, restricting alcohol consumption, maintaining healthy body weight, increasing regular aerobic physical activity and adopting a healthy plant-based diet with limited sodium intake.^21^ Secondary prevention would be targeted to at-risk individuals to adequately treat their diseases to prevent them from suffering a stroke – ensure good blood pressure control, treat with a statin and aspirin or anticoagulants such as warfarin and rivaroxiban, if indicated in atrial fibrillation.^35^ It is vital to have HIV-positive patients on antiretroviral treatment to suppress their viral load. Tertiary prevention would target the clinical and outcome stages of the disease, such as a dedicated stroke unit with the facilities to thrombolyse patients with an acute ischaemic stroke.^28,35^ Finally, quaternary prevention, as described by the World Organization of Family Doctors (WONCA) International Dictionary for General/Family Practice, can be interpreted as activity taken to detect patients at risk of overmedication, to safeguard them against additional medical intrusion and to recommend interventions that are morally acceptable.^36^ This principle can also be linked to the ethical concept of ‘Do no harm’, as medical intervention may lead to unintended sequelae.

### Patient outcomes

Stroke survivor outcomes were measured in this study using the MRS^13^ and are discussed and compared in Table 2 with the outcomes that were found in the 2008 study by Bertram et al.^10^ on South African stroke survivors.

**TABLE 2:** Stroke survivor outcomes: Comparison between this study and Bertram et al.^10^

Outcome	Findings from this study (*n* = 15) (%)	Findings from Bertram et al.^10^ (%)
No disability	26.7	2.0
Symptoms with no disability	20.0	40.0
Slight disability	6.7	23.0
Moderate disability	13.3	12.0
Moderately severe disability	20.0	15.0
Severe disability	13.3	8.0

Serious neurological sequelae for patients who survived their strokes are still a major problem at NDH. There are clear, published, cost-effective guidelines^14,15,19,20,28^ available, which can significantly improve outcomes, but need stringent implementation and application. Factors limiting the clinicians’ ability to improve patient care include clinical governance issues such as poor record-keeping, poor healthcare planning and a lack of priority patient transport.

Regarding the final diagnoses made for the patients who presented with signs and symptoms suggestive of stroke to the emergency department, 59.9% were diagnosed with an ischaemic stroke and 22.7% had transient ischaemic attacks, which is roughly in keeping with previous studies on the topic. Haemorrhagic stroke accounted for 13.6% of patients and 4.6% were classified as ‘other’. These results align well with other published data, which puts the prevalence of haemorrhagic strokes at 10% – 15%.^7,37,38^ The risk factor that most prominently contributed to a haemorrhagic stroke was hypertension, with 66.7% of patients who had a haemorrhagic stroke also having previously diagnosed with hypertension. In addition, 16.7% of patients with a haemorrhagic stroke had HIV as the only risk factor.

This study’s strengths lie in the detailed data that were collected, but it is limited by the study size. The study size was primarily limited by poor record-keeping practices as less than half of the files could be retrieved. The hospital still uses a paper-based filing system. The study was also limited by its retrospective nature. Because of the limited amount of data that could be retrieved as well as missing data, misclassification bias is a distinct possibility.

## Conclusion

Stroke remains a significant clinical and public health concern in Bloemfontein and the broader South African population. Improving patient outcomes should be a high priority for the Free State Department of Health to adhere to international standards of practice. All these problems trickle down to impact patient outcomes. Establishing a dedicated stroke unit at NDH is just one of the ways to improve the lives and livelihoods of patients affected by the devastating consequences of cerebrovascular accidents but should not be the only way we strive to improve our healthcare delivery.

### Recommendations

This study underlines the importance of stroke and cardiovascular disease prevention and stresses the value of establishing dedicated stroke units in South Africa, particularly at NDH. Future research could explore outcomes after the intervention (such as applying dedicated stroke protocols) was applied and the role that EMS plays in patient outcomes when presenting with medical emergencies, such as a stroke.
